# Optimal adjuvant therapy in older (≥70 years of age) women with low-risk early-stage breast cancer

**DOI:** 10.1038/s41523-023-00591-6

**Published:** 2023-12-14

**Authors:** M. Chadha, J. White, S. M. Swain, E. Rakovitch, R. Jagsi, T. Whelan, J. A. Sparano

**Affiliations:** 1 Department of Radiation Oncology, Icahn School of Medicine at Mount Sinai, New York, NY USA; 2 Department of Radiation Oncology, University of Kansas Medical Center, Kansas City, KS USA; 3 Department of Medicine, Georgetown Lombardi Comprehensive Cancer Center, MedStar Health, Washington, DC USA; 4 Department of Radiation Oncology, Sunnybrook Health Sciences Centre, University of Toronto, Toronto, ON Canada; 5 Department of Radiation Oncology, Winship Cancer Institute, Emory University, Atlanta, GA USA; 6 Division of Radiation Oncology, Department of Oncology, McMaster University and Juravinski Cancer Centre at Hamilton Health Sciences, Hamilton, ON Canada; 7 Division of Hematology and Medical Oncology, Department of Medicine, Tisch Cancer Institute, Icahn School of Medicine at Mount Sinai, New York, NY USA

**Keywords:** Breast cancer, Breast cancer

## Abstract

Older women are under-represented in breast cancer (BC) clinical trials, and treatment guidelines are primarily based on BC studies in younger women. Studies uniformly report an increased incidence of local relapse with omission of breast radiation therapy. Review of the available literature suggests very low rates of distant relapse in women ≥70 years of age. The incremental benefit of endocrine therapy in decreasing rate of distant relapse and improving disease-free survival in older patients with low-risk BC remains unclear. Integration of molecular genomic assays in diagnosis and treatment of estrogen receptor positive BC presents an opportunity for optimizing risk-tailored adjuvant therapies in ways that may permit treatment de-escalation among older women with early-stage BC. The prevailing knowledge gap and lack of risk-specific adjuvant therapy guidelines suggests a compelling need for prospective trials to inform selection of optimal adjuvant therapy, including omission of adjuvant endocrine therapy in older women with low risk BC.

## Introduction

Annually, 268,600 women are diagnosed with breast cancer (BC) in North America, and of these, 30% are ≥70 years of age^1^. The majority present with early-stage disease and are eligible for breast conservation surgery (BCS)^2,3^. SEER data on the US Incidence of breast cancer subtypes by age note that majority BC among women between the age of 65–74 and ≥75 years had favorable subtype (receptor positive/HER 2 negative), 77.8% and 80.1%, respectively^4^. Further, triple negative and HER2 positive subtypes were less likely to be diagnosed in women over the age of 65 years.

The EBCTCG meta-analysis^5^ on breast radiation therapy (RT) included 10,801 women with BC derived from 17 randomized trials. The analysis demonstrates that RT reduced the rates of first loco-regional relapse (LR) and/or distant relapse (DR) by 54% (HR 0.46, 95% confidence interval (CI) 0.41–0.51, *p* < 0.0001). The 10-year risk of any first recurrence decreased from 31.0% to 15.6% (95% CI 13.2–17.6, 2*p* < 0.00001). RT reduced BC death by about a sixth (RR 0.83, 95% CI 0.73–0.95), and the 15-year risk of BC death decreased from 20.5% to 17.2% (95% CI 0.8–5.8, 2*p* = 0.005). Specifically, among the 1340 women ≥70 years the benefit of RT was similar (HR 0.47, 95% CI 0.33–0.66), the 10-year risk of any first recurrence LR or DR reduced from 17.7% vs. 8.8% (2*p* = 0.00002), including the subset of women with estrogen receptor-positive (ER+) T1 intermediate grade tumors. Contemporary RT standard of 1-week to 3-week duration is associated with reduced treatment burden and toxicity^6–14^. The implications that patients experience increased inconvenience and RT-related treatment burden are notably in reference to an outdated 5–7 weeks fractionated RT schedule.

Similarly, the benefit of endocrine therapy (ET) in reducing recurrence risk in ER + BC is established through numerous clinical trials^15–20^. The meta-analysis reported on 88,023 women with BC, including 66,408 with N0 disease (75.4%). Comparing 5 years of tamoxifen versus none, the updated analysis noted 39% reduction in recurrence risk (HR 0.61, 2*p* < 0.0001) with tamoxifen. This translated into a reduction in the annual event rate in years 0–15 from 4.7% to 3.0%^19^. These data and results from several clinical trials showing the benefit of ET^14–19^ drives our current guidelines for recommending ET × 5 years for ER + BC patients^21^.

Of note, many past clinical trials, and as such the meta-analysis included very few women ≥70 years^16,22^. Only 3972 (4.5%) women were ≥70 years in the meta-analysis. In this group, the annual event rate reduced from 5.7% vs. 3.3% (HR 0.49, SE 0.12) with tamoxifen. The rates of non-BC mortality far exceed BC-specific mortality in older women; thereby confounding the absolute clinical benefit of ET on all-cause mortality in this population.

## Adjuvant endocrine therapy (ET) with omission of radiation (RT) in older women

Prior to the availability of molecular genomic assays (MGA), Phase III trials including, CALGB 9343, PRIME II, Canadian, and ABCSG 8A evaluated the omission of RT in post-menopausal women^23–28^ (Table 1). In all four studies, the randomization was between RT + ET versus ET alone.

**Table 1:** Randomized trials evaluating post-lumpectomy radiation benefit in older patients.

Study	Inclusion age criteria (years)	T-size	Randomization	# patients	Local relapse (%) *p* value; F/U years		Distant relapse (*n* = no. of patients) or (%)		Overall survival (%) *p* value; F/U years	
					ET + RT	ET	ET + RT	ET	ET + RT	ET
CALGB 9343 *JCO 2013*	≥ 70 years	≤ 2cm	ET vs ET + RT	636	2%	10%	*n* = 17	*n* = 10	67%	66%
					*p* < 0.001; 10-years		10-years		*p* = 0.64; 10-years	
ABCSG 8A Trial *Int. J. Rad. Biol Phy 2007*	≥ 50 years	< 3cm	ET vs ET + RT	831	2.5%	7.6%	*n* = 16	*n* = 12	86.6%	87.6%
					*p* = 0.0004; 10-years		10-years		No difference *p* = 0.18; 10-years	
Canadian Trial *NEJM 2004*	≥ 50 years	≤ 5cm	ET vs ET + RT	769	0.6%	7.7%	4.5%	4%	93.2%	92.8%
					*p* = <0.001; 8-years		8-years		No difference *p* = 0.69; 8-years	
PRIME II *NEJM 2023*	≥ 65 years	≤ 3cm	ET vs ET + RT	1326	0.9%	9.8%	0.5%	1%	81%	80.4%
					*p* = 0.0008; 10-years		5-years		No difference (*p* = 0.34; 10-years)	

Between 1994 and 1999, the CALGB 9343 trial^22^ randomized 636 women, ≥70 years old, with stage T1, node-negative (N0) ER + BC. RT was delivered over a 6.5-week duration (33 fractions). The 10-year LR rate with RT + ET and ET alone was 2% and 10%, respectively (*p* < 0.001). The overall risk of Distant Relapse (DR) was low. Specifically, 27 patients developed a DR, 10 in the RT + ET arm, and 17 in the ET arm. At a median follow-up of 12.6 years, only 21 BC deaths were reported (1% in each group), and majority of the 334/636 women had died from non-BC causes.

Between 2003 and 2009, the PRIME II trial randomized 1326 eligible women ≥65 years old with ER + BC, T-size ≤3.0 cm, and pN0^23,24^. RT was delivered over 5-week duration (25 fractions). At a median follow-up of 10 years, Local relapse (LR) rates with RT + ET and ET alone was 0.9% and 9.8%, respectively (*p* < 0.0008). DR was observed in only 5 patients in RT + ET and 4 patients in ET only group. Majority of the deaths (87%) were from non-BC causes.

Between 1992 and 2000, the Canadian Trial^25^ randomized 769 women age ≥50 years, with ER + BC, T-size ≤5 cm, N0. The 5-year LR was 0.6% in ET + RT arm and 7.7 % in ET alone arm (*p* < 0.001). The disease-free survival was 91% and 84% in the ET + RT and ET alone group, respectively (*p* = 0.004). No differences in rate of DR (*p* = 0.69), and BC deaths reported was the same (*n* = 10) in each group.

Between 1996 and 2004, the ABCSG trial 8A randomized 869 postmenopausal patients ER + low-risk BC^26,27^. RT was delivered over 6 weeks (25 fractions + boost). At a median follow up of 9.8 years, the rate of LR in the RT + ET and ET alone groups was 2.5% and 7.6%, respectively (*p* < 0.0004), and DFS was 94.5% and 88.4%, respectively (*p* = 0.015). Overall, 28 patients developed DR, 16 patients in the RT + ET group, and 12 patients in the ET alone group.

While expecting modestly higher rates of LR with omission of RT, the risk of DR and BC mortality is very low in favorable risk BC. Data from these trials culminated in guideline recommendations to offer older (≥70 years) women with stage I ER + BC the option to omit breast RT when prescribing ET.

### Consequences of local recurrence

Management of subsequent ipsilateral breast tumor recurrence (IBTR) requires additional surgery and adjuvant treatment. With initial omission of RT, further BCS + RT is an endorsed option for treatment of IBTR. However, available data on management of IBRT from the CALGB and PRIME II trials^22,23^ shows that almost 50% of older women undergo mastectomy. In the aging population, IBTR is not without risk given the psychological distress from repeat cancer events, and the complex healthcare needs as women are older and possibly frail^29^.

## Studies using MGA-defined risk groups in ER + BC

MGA has improved prognostication and risk stratification of BC compared to clinic-pathological features only, and with some assays we gain predictive information to safely omit chemotherapy^30–33^. The utilization of MGA in older BC patients may further help refine risk-tailored algorithms for adjuvant ET.

Table 2 summarizes rates of LR, DR and BC-specific mortality in women ≥70 years categorized by 21-gene Oncotype DX Recurrence Score (RS)™. The Clalit Health Services registry^34^ reported a higher proportion (55%) of women ≥70 years had low RS < 18 compared to younger women (45%), *p* = 0.004. The 5-year Kaplan–Meier estimates of DR among the 181 women ≥70 years with RS < 18 was 0.6%, and BC-specific mortality rate was 0.4%. Similarly, SEER study identified 5988 women ≥70 years of age with available RS. Among the 3424 women with pN0 and low RS (<18), and the 5-year rate of BC-specific mortality was only 1.3%^35,36^.

**Table 2:** Distant relapse rates in low-risk breast cancer.

Study	Number of patients	Median age	Genomic assays Low-risk profile	ET	Distant relapse
Tailor Rx *NEJM 2015, 2018*	1626	58 years	Oncotype RS < 11	Yes	FFDR rate 99.3% @ 5 years and 96.8% @ 9 years
MINDACT study *JCO 2022*	741	55 years	MammaPrint ultralow risk/clinical low risk	No Yes	DMFI rate 97.8% @ 8 years DMFI rate 97.4% @ 8 years
RASTER Study *Eur. J. Cancer; 2022*	140	50 years	MammaPrint low- risk/clinical low-risk	No	DRFI rate @ 10 years is 93.9%
Clalit Health Services Registry *npj Breast Cancer 2017*	381	≥ 70 years	Oncotype RS < 18	Yes	KM estimates DR rate @ 10 years is 0.6%

*FFDR* Freedom from recurrence at distant site, *DMF*I distant metastases-free interval, *DRFI* distant recurrence-free interval, *KM* Kaplan Meier, *DR* distant relapse.

The TAILOR Rx study^37,38^ included 1619 women (only 113 were ≥70 years) with low RS (0–10) treated with ET alone. At 5 years, the rate of freedom from recurrence at distant site in this favorable low-risk cohort was 99.3% (95% CI, 98.7–99.6).

The EORTC 10041/BIG 3-04 MINDACT phase 3 trial^39^ enrolled 6693 women <70 years. In an exploratory analysis of clinical–low and genomic–ultralow-risk BC, the distant metastases-free interval (DMFI) was 97.6%, and there was no difference in DMFI between patients receiving no adjuvant treatment (NAT) and ET alone, 97.8% and 97.4%, respectively.

The RASTER^40^ prospective community-based observational study defined risk groups using the 70-gene signature (MammaPrint)™ risk and Adjuvant! Online (AOL). In 140 clinically/genomic identified low-risk patients who did not receive any systemic therapy, the reported 10-year rates of the distant relapse-free interval (DRFI) was 93.9% (95% CI 90.0–98.1), and BC-specific survival was 96.7% (95% CI 93.5–99.9).

It is noteworthy that the study design of all ongoing trials^41–45^ using MGA to define low-risk BC, across the board prescribe ET. Hence, despite contemporary MGA trials outcomes with omission of ET in low-risk BC will remain unaddressed.

From the summarized data, MGA regardless of stage or grade is able to identify older BC patients with extremely low rates of DR. In patients with low-risk of DR, the absolute benefits of reduction in relapse anticipated with ET similarly may be small as well. As such, the health risks and side effects may outweigh the benefits. If this relationship is indeed borne out, with prospective trials designed to test this question in women ≥70 years of age, ET may constitute overtreatment.

## Adjuvant comprehensive local therapy alone with omission of endocrine therapy

There are very few randomized studies on early stage BC that includes a treatment arm with omission of ET^46–48^ (Table 3). Of note, all these studies predate the practice of integrating MGA.

**Table 3:** Trials Evaluating tamoxifen benefit on node negative, breast cancer all ages.

	Total # of patients	Age ≥ 70 years # of patients	Randomization	Disease-free survival	Overall survival
NSABP B14 *N. Eng. J. Med. 1989*	2817	0	ET vs No ET	*P* < 0.00001	No diff: in >50 yrs. *P* = 0.13
BASO II *Eur. J. Cancer 2013*	1135	0	ET vs RT vs RT + ET vs Observation	NR	No diff
GBSG-V *Eur. J. Cancer 2010*	347	NR	ET vs RT vs RT + ET vs Observation	No diff	No diff
NSABP B21 *JCO 2002*	1009	159	ET vs RT vs RT + ET	No diff *P* = 0.28	No diff *P* = 0.93

*NR* not reported.

Between 1982 and 1987, the NSABP B14 study^21^ randomized 2644 women <70 years with ER + N0 BC to receive tamoxifen + RT, and placebo + RT. The mean age of the study subjects that reported better DFS and no difference in OS (*p* = 0.13) with tamoxifen was 55 years. There is caution when translating an observed benefit in largely younger women to older ≥70 year women.

Between 1992 and 2000, the British Association of Surgical Oncology (BASO) II trial ^45^ enrolled 1135 women <70 years with ER + BC, stage T1N0, grade 1 or good prognostic subtype. In a 2 × 2 factorial design treatment arms, included surgery alone, ET alone, RT alone, and RT + ET. The highest LR was with surgery alone at 2.2% per annum, and there was no difference in LR and BC-specific survival in women treated with S + RT alone and S + ET alone.

From 1991 to 1998, the German Breast Study Group Trial V (GBSG-V)^46^ study with similar design as the BASO trial enrolled 361 patients between the ages of 45 and 75 years with stage T1N0 BC. At a median follow-up of 9.9 years, the reported LR rates in the surgery alone, ET alone, RT alone, or RT + ET arms were 34%, 10%, 13%, and 7%, respectively. No difference in the event-free survival was noted between S + RT alone and S + ET alone groups.

Between 1989 and 1998, the NSABP B-21 trial^47^ enrolled 1009 women with ER + BC, T-size <1 cm, N0 BC. Following BCS, the randomization included RT alone, tamoxifen alone, or RT + tamoxifen. A statistically significant difference in LR (*p* = 0.008) is noted between the 3 arms. With median follow-up of 86.9 months, no difference in rates of DR (*p* = 0.28) or OS (*p* = 0.93) was reported. In this largely low-risk study population, the rate of DR in RT only (3.2%), ET only (3.3%), and RT + ET (1.5%) groups was similar. The data showed a small number of BC deaths across treatment arms (*n* = 9 Tam; *n* = 8 RT; *n* = 5 ET + RT).

The Ontario population-based study^49^ included 5076 women age ≥65 years with T-size ≤2 cm, N0, ER + BC. The 5-year rate of DR for RT + ET was 0.43%, ET alone 0.71%, RT alone 0.71%, and 1.7% in patients receiving NAT. The 5-year OS by treatment was 95.7% RT + ET, 81.6% ET alone, 92.8% RT alone, and 71.5% NAT (*p* < 0.0001).

From the available randomized and population-based data, the omission of ET in early-stage low-risk BC does not signal any detrimental shifts in disease outcomes including change in rates of DR with omission of ET.

## Contralateral breast cancer (CBC) risk in older women

Available data shows that age at initial diagnosis is the most important predictor of subsequent risk for CBC, and this risk exponentially decreases with increasing age of primary BC diagnosis^50–52^. Therefore, one might surmise that CBC risk is low in women ≥70 years age at diagnosis. If benefits of ET in reducing risk of CBC were demonstrably limited, older women might well desire to forgo ET and its associated negative impact on quality of life (QoL).

## Endocrine therapy and quality of life

Most commonly, older postmenopausal patients are prescribed AI. However, there is an increased incidence of AI-associated bothersome skeletal events and musculoskeletal symptoms, and the rates of non-adherence to AI approaching 25–30% is not trivial^53,54^. Older women have a high prevalence of underlying health issues and comorbidities, which make them more susceptible to tolerating ET poorly^55–61^ (Table 4).

**Table 4:** Compliance to therapeutic course of endocrine therapy.

Author	# patients	Patient group	Non-compliance	Contributing factors
He et al., *JCO 2015*	3395 Survey	Pre and postmenopausal	14%@ 1 year 36% @ 3 years 54% @ 5 years	Older age Comorbidity Need analgesics & sedatives
Salgado et al., *Br. C. Res Treat 2007*	622 Survey	Postmenopausal	30%	84% because of adverse effects
Sheppard et al., *JCO 2014*	1288 Interviews	≥ 65 years	51.5%	Frail Health Older age Race, Stage
Hirshman et al., *JCO 2010*	8769 Database	42% ≥ 65 years	51% @ 4.5 years	Age (older and younger groups) Higher comorbidity

Prospective PRIME trial QoL studies^62^ illustrate different toxicity profiles ET and RT. RT-related breast symptoms markedly improve by 15 months after completion of RT, while ET-associated side effects persist over the 5-year duration of ET therapy. The prospective CANTO trial studied 4262 BC patients (63% were post-menopausal). The study results note that in postmenopausal women, ET had a differentially worse and persistent effect on QoL at 2 years from diagnosis^63^.

The ET-associated cognitive decline with a negative impact on verbal learning/memory, visual learning/memory, frontal executive function, and processing speed is particularly of concern in an aging population^64–67^.

In summary, ET is associated with negative impact on QoL and high rates of poor tolerance of ET. The balance between benefit and risks of ET in women with low-risk BC is not well defined. There is compelling rationale for pursuing clinical trials in older women that will provide unequivocal evidence on the absolute therapeutic benefit of ET in low-risk BC, and help drive risk-tailored recommendations for adjuvant ET.

## Role of comprehensive geriatric assessment (CGA)

In clinical trials and routine clinical practice, the importance of including CGA is being strongly promoted^68–72^. In the aging population, CGA is a helpful measure for stratifying individual risk, and may guide selection of therapeutic options according to biological age, functional status, intrinsic capacity, frailty, possible resilience, comorbidities, polypharmacy, and patient preferences. Alongside validated QoL assessments, prospective clinical trials should include CGA in study of older cancer patients. With such informed assessments, CGA may emerge as the one of the important determinants for establishing optimal adjuvant therapy in breast cancer.

## Future directions and ongoing studies

To define risk-tailored treatment based on absolute clinical benefit by type of adjuvant therapy for early stage BC in older women requires modern prospective trials. The current NCCN guidelines and practice will not change without level 1 evidence demonstrating low, comparable event rates with improved QoL among older women with ER+ early BC treated with BCS + RT alone.

Two ongoing studies seeking to optimize the approach to adjuvant RT and ET in older women with early-stage BC are open and accruing patients in Italy and Canada. There are currently no trials in this patient population in the US (Table 5).

**Table 5:** Ongoing Clinical Trials.

	EUROPA *N* = 926	REaCT trial *N* =100
Inclusion criteria	Women Aged ≥70 Years with Luminal A-like tumors (defined as ER ≥ 10%, PR > 20%, HER2 negative, with Ki67 < 20%)	Women > 70 years with grade 1 T size ≤ 5 cm, grade 2 T-size ≤ 3 cm, or grade 3 T-size ≤ 1 cm. N0, ER + HER2-
Randomization	Lump + Endocrine therapy (ET) vs Lump + PBI alone	Lump or mastectomy + ET x 5 years vs Lump or mastectomy + No ET
Endpoint	**Primary Endpoints:** • HRQol using the EORTC QLQ-C30 • Time to ipsilateral breast tumor recurrence **Secondary Endpoints:** • HRQoL EORTC B23, QLQ ELD14 • Locoregional relapse, contralateral • Distant relapse, • Breast cancer specific survival • Overall survival • Adverse events • Cosmesis	**Primary Endpoints:** • Accrual of 100 participants across 8 centers within 2 years after study initiation • A participation rate of at least 60% among participants approached. • At least 90% of enrolled participants receive treatment as per their allocated intervention for at least 4 weeks **Secondary Endpoints:** • Significant adverse events • ET related Toxicity • FACT B- Endocrine Symptom Subscale (FACT-B plus ES). • CARG patient tool

Exclusive Endocrine Therapy Or Partial Breast Irradiation for Women Aged ≥70 Years With Luminal A-like Early Stage Breast Cancer (EUROPA Trial—NCT04134598) study at the University of Florence opened to accrual since 2021 and plans to enroll 926 patients. Eligible patients have clinical low-risk hormone-sensitive BC, and randomization is between partial breast radiation (PBI) or ET alone. In comparing PBI to ET, the primary study endpoints include health-related QoL using the EORTC QLQ-C30, and non-inferior local recurrence rates. An interim analysis is planned when 152 enrolled patients reach the 2-year follow-up. Stopping rules include a >2% IBTR rate per year or a >7% DM rate at any time. Although this study will generate important evidence, history suggests that practice in the US is unlikely to change in the absence of a trial conducted in North America, given differences in co-morbidities, medical practice patterns, and patients’ values and preferences.

Evaluating Harms and Benefits of Endocrine Therapy in Patients ≥70 Years of Age with Lower Risk Breast Cancer (REaCT trial—NCT04921137), study sponsored by Ottawa Hospital Research Institute opened to enrollment in 2021. The study aims to evaluate the feasibility of ET omission in women ≥70 years treated with standard local therapy. The randomization is between no ET vs ET for 5 years. The study will enroll 100 patients with low-risk BC categorized by histopathologic features only. The primary outcomes include assessment of adverse events/toxicity and health-related QoL.

## Conclusions

Women ≥70 years constitute a distinct group of BC patients with rising incidence of early stage BC amenable to BCS. The widely accepted standard of 1–3 week short-duration RT schedule is associated with less clinical and financial toxicity. Comprehensive local therapy (BCS + RT) is well tolerated, and beneficial in reducing risk of the most common site of BC relapse observed in older (≥70 years) women. Breast RT remains a relevant adjuvant option in our aging BC population.

Better understanding of the clinical–pathologic factors and MGAs have improved our ability to classify low-risk ER+, HER2-negative BC across all age groups. Older patients with favorable-risk BC treated with ET have very low rates of DR. As such, with the omission of ET, the up to 50% anticipated increased risk of DR in this population might remain low and clinically acceptable. To date, there is very little research aimed at defining age-specific recommendations for adjuvant ET in women ≥70 years.

To inform future practice guidelines for clinicians and patients, we advocate pursuing prospective trials to establish optimal age-adjusted, risk-tailored adjuvant therapy in older BC patient population, including systematic geriatric assessment, and the role of comprehensive local therapy alone with omission of ET as an alternative.
